# Promoting agricultural entrepreneurship through practical education in Chinese universities

**DOI:** 10.1371/journal.pone.0340670

**Published:** 2026-02-10

**Authors:** Wei Hou, Huajian Song, Qianwen Yang, Lei Luo, Jingyi Ding, Yipei Cao, Ruihua Hu, Guo Li, Hai Ma

**Affiliations:** 1 College of Marxism, Sichuan Agricultural University, Chengdu, Sichuan, China; 2 Zhejiang Institute of Administration, Hangzhou, Zhejiang, China; 3 College of Water Conservancy and Hydropower, Sichuan Agricultural University, Chengdu, Sichuan, China; 4 College of Economics, Sichuan Agricultural University, Chengdu, Sichuan, China; 5 Chongzhou Institute of Administration, Chengdu, Sichuan, China; Instituto Tecnologico Autonomo de Mexico, MEXICO

## Abstract

The social phenomenon of university students learning agriculture rather than pursuing it, understanding agriculture rather than choosing agriculture, is a major challenge for the sustainable development of agricultural talents in China. This research paper investigates the impact and mechanism of practical education in agricultural schools on students’ intention to start agricultural businesses using OLS, Probit, and mediation effect models. Data from a survey of 1612 Chinese students from Sichuan Agricultural University, Nanjing Agricultural University and other 6 agricultural universities was analyzed, with instrumental variable probit model (IV-Probit) utilized to tackle endogeneity issues. The results showed that (1) The implementation of rural social practice, volunteer service, entrepreneurship competition, and agricultural labor education by universities can significantly boost university students’ interest in starting their agricultural businesses. Additionally, for every additional type of practical education students receive, they are 11.0% more likely to want to start business in agriculture. (2) Practical education will affect individual entrepreneurial effectiveness perception, including economic benefit perception, social status perception and professional value perception. Among these, the mediating effect of professional value perception is the strongest, with a ratio of 16.1%. (3) The results suggest that entrepreneurship support policy play a role in moderating the connection between individuals’ economic benefit, social status perceptions and their intentions. (4) The intentions of university students to start agricultural businesses are significantly correlated with their control variables, including major selection, students cadre experience, agricultural cognition, household registration, and family members’ occupation. Therefore, it is suggested that there should be a focus on enhancing practical education in agricultural universities, fostering students’ confidence in agricultural entrepreneurship, and optimizing sustainable policy support for university students’ entrepreneurial endeavors.

## 1. Introduction

The involvement of university students in rural undertakings has the potential to inject new vitality into the process of agricultural modernization [1]. On the other hand, there has been a notable rise in the employment demands faced by university students, leading to the agricultural sector becoming a crucial source for providing job opportunities to young individuals pursuing higher education. In 2021, the Ministry of Agriculture and Rural introduced a national model for rural return and entrepreneurship, while also promoting agricultural business opportunities for young university students. In recent years, Central Document No. 1 has also explicitly stated the strengthening of rural talent team construction through the “Rural Revitalization Talent Support Program”. In a similar vein, it is anticipated that the number of graduates from Chinese universities across the nation will reach 11.79 million in 2024, marking an increase of 210,000 compared to the previous year. The continuously increasing number of graduates and the increasingly fierce job competition environment have led to a spiraling rise in the employment difficulties faced by university students. Therefore, both the government’s work report and the Chinese Conference on Entrepreneurship for College Graduates stressed the need to boost rural employment for university students and offer support services for rural revitalization. This indicates that guiding university students in an organized way to pursue agricultural employment and entrepreneurship has become a viable strategy to alleviate current employment challenges and expedite the modernization of agriculture. A comprehensive examination shows that many countries have introduced various programs to promote youth agricultural entrepreneurship. Specifically, Germany and Austria have nurtured students’ entrepreneurial tendencies by creating a strong entrepreneurial environment and providing effective support systems [2].

Like many developing countries, Chinese young people have an inherent fear and aversion towards agriculture [3]. When they first enter society, their lack of skills and knowledge makes it difficult for them to engage in agricultural production and business activities. It is regrettable that the current lack of strong entrepreneurial intentions among university students in agriculture is widespread [4]. The 2020 China Census Yearbook reveals that only 13.6% of the population under the age of 35 who work in agriculture, forestry, animal husbandry, and fisheries are employed or involved in entrepreneurship. This figure ranks as the lowest among the 20 industries surveyed, significantly lagging behind the average of 32.9%. According to the data released by the Ministry of Education in 2023 in the “2023 Report on Employment of Chinese Undergraduates”, Only 1% of the 2022 graduates are employed in the agricultural, forestry, animal husbandry, and fishing industries. Maikosi Data has found that around half of agricultural students come from farming families. Despite having the advantage of agricultural entrepreneurship and employment, only 55% of undergraduate agricultural students are working in a field related to their major six months after graduation. Various sectors of society generally believe that there should be a stronger emphasis on the application and transformation of agricultural science sub-disciplines.. Given the historical mission of agricultural universities to “cultivate talents of agriculture” and the high professional standards of agricultural students, the pressing issue of guiding young university students towards changing their internal perceptions and fostering agricultural entrepreneurship has emerged as a prominent topic in agricultural science, technology, economic and sustainable development. As the primary hub for nurturing talents in modern agricultural development, agricultural colleges play a crucial role in advancing the progress of agriculture, rural prosperity, and the financial well-being of farmers [5]. By participating in their professional agricultural education programs, young students can enhance their professional knowledge, develop agricultural entrepreneurship skills, and increase their chances of finding employment. Practical education, as a crucial component of talent development in higher education institutions, is instrumental in molding students’ values, fostering innovation and entrepreneurship, and promoting a spirit of service to society [6–8]. So, is it possible for practical education programs in agricultural colleges to motivate university students to start agricultural businesses, ultimately benefiting both youth employment and the revitalization of rural talent? This issue has not yet been verified at the empirical level. Can practical education activities categories such as social practice, volunteer service, entrepreneurship competition, and agricultural labor education enhance university students’ motivation to participate in agricultural entrepreneurship? What are the effects and mechanisms of its influence? Does policy support have a regulating effect on the entrepreneurial intentions of university students? Research has been unable to provide a definitive answer. Hence, this research is founded on survey responses gathered from 1,612 agricultural students enrolled in six Chinese agricultural universities. We employ OLS, probit, and mediation effect models to validate the impact and mechanism of practical education on students’ readiness to start agricultural businesses in agricultural universities. We also examine the mediating transmission and moderating effect paths involved in the process. Using the instrumental variable method to address endogeneity issues, with the aim of providing recommendations for universities and relevant government departments in promoting agricultural entrepreneurship among university students.

## 2. Theoretical analysis and research hypothesis

Entrepreneurial intentions of individuals serve as a reliable indicator of their future entrepreneurial behavior and play a crucial role in forecasting upcoming entrepreneurial activities [9,10]. Numerous researchers have discovered that personal factors such as gender, age, level of education, income expectations [9,11], personal interests, employment concepts [12,13], among others, play a significant role in shaping individual agricultural entrepreneurial employment intentions. The significant impact of household factors such as household registration type, economic conditions, family cultural capital, and family involvement in agricultural employment on individuals [14,15]. External environmental factors primarily consist of factors such as local location factors, entrepreneurship support policies [16], social support, and agricultural infrastructure [17,18]. The primary focus of agricultural entrepreneurship research is on three key groups: farmers, migrant workers, and young students [19]. While there is a wealth of research on the entrepreneurial challenges and pathways of farmers and returning migrant workers in agriculture [20,21], studies on students entrepreneurship in this field are limited [22,23]. In addition, university students have strong adaptability and great flexibility, which sets them apart from traditional entrepreneurship research fields [24]. The heterogeneous and significant intention of this group in agricultural entrepreneurship is distinct [25]. Especially given the current employment landscape, it is important to address, assess, and delve into the topic of agricultural entrepreneurship among recent graduates [26].

### 2.1. Impact of practical education in higher education on university students’ inclination towards agricultural entrepreneurship

Some scholars are focusing on university students as the main body of agricultural entrepreneurship, and they have found that, based on the factors influencing individual resource endowments mentioned above, the impact of higher education cannot be ignored. A study comparing traditional and non-traditional agricultural disciplines revealed that students who studied traditional agricultural disciplines were more prone to developing an interest in agricultural entrepreneurship [27]. Moreover, research has shown the importance of hands-on learning, such as the rural social practice, in promoting agricultural innovation and entrepreneurship education. It is suggested that there should be a seamless integration between theoretical classroom learning and practical social practice to enhance students’ educational experience [28]. The organisms in nature are complex and diverse [29], which determines that a greater knowledge reserve is required for engaging in agricultural work. To cultivate well-rounded agricultural talents, the entrepreneurship education system for students needs to adopt multiple approaches. It is easy to understand how hands-on education at universities can play a significant role in enhancing the ability of young university students to identify opportunities and inspire them to start agricultural businesses. Although there have been comprehensive studies analyzing the individual, family, and environmental factors that influence university students’ agricultural entrepreneurship, as well as the role of practical education in this process, there is a lack of more in-depth and detailed categorization and exploration. Chinese Ministry of Education has issued the “Opinions on the Plan for Cultivating Outstanding agricultural Talents”, emphasizing the need to strengthen practical education for agricultural students. It aims to guide students to learn about agriculture, love agriculture, and understand the importance of agriculture. Students are encouraged to write their theses on the land of the motherland, in order to fully enhance their sense of mission and responsibility towards serving rural and the modernization of agriculture and rural areas. But there has been no research on the dimensions and impact of practical education on students entrepreneurship. This presents an opportunity for innovative exploration.

Therefore, the hypothesis H1 suggests that practical education, including university students’ participation in rural social practice, volunteer service, entrepreneurship competition, and agricultural labor educations, positively influences their willingness towards agricultural entrepreneurship.

### 2.2. University students’ willingness to start agricultural business and its correlation with entrepreneurial effectiveness perception

Some studies have also made progress in understanding the mechanisms of influence. Some scholars have further explored the moderating effect of policy understanding cognition on the relationship between park environment and agricultural entrepreneurial intention [16]. Furthermore, scholars examining the relationship between social capital and university students’ entrepreneurial intentions have found that individual entrepreneurial self-efficacy is a crucial mediating factor in this process [17]. In addition, some scholars have further applied self-efficacy theory to suggest that entrepreneurship education promotes the willingness of individual students to start agricultural businesses by increasing their perception of self-efficacy [19]. Cognitive theory suggests that individuals develop personal understandings and perspectives of their environment and surroundings, translating their senses into cognition that ultimately shape their behavioral intentions on a psychological level [30]. With the development of cognitive theory and its entry into the entrepreneurial field, the concept of entrepreneurial efficacy has begun to rise in the academic circle. This concept pertains to an individual’s personal belief in their capacity to achieve a goal and the rewards it entails. Through the acquisition, encoding, storage, and retrieval of information related to the goal, individuals ultimately develop a strong belief in their ability to successfully accomplish it. As research continues to expand, some studies suggest that entrepreneurial self-efficacy can increase the success rate of entrepreneurship and effectively promote individual entrepreneurial intentions [31,32]. A research study involving university students who have encountered entrepreneurial setbacks revealed that enhanced entrepreneurial self-efficacy can assist in cognitive restoration and motivate individuals to engage in re-entrepreneurial activities [33]. Research findings also confirm that individuals with high levels of self-efficacy are more willing to try entrepreneurship and challenge themselves, especially those who have a positive assessment of the potential benefits of entrepreneurship [34]. There is a theory that divides entrepreneurial efficiency perception into economic benefits perception, social status perception, and personal value perception [19]. In addition, it should also be seen that young people with specific agricultural skills have a professional value perception for the industry with which they are familiar. Through practical education in university, students experience a notable increase in their entrepreneurial effectiveness perception, specifically in terms of perceived economic benefit, social status, and professional value. This heightened entrepreneurial effectiveness perception ultimately leads to a greater inclination towards engaging in agricultural entrepreneurship, highlighting the mediating role of entrepreneurial effectiveness perception in this context.

The following hypotheses are formed:

H2a: Economic benefit perception plays an intermediary role in influencing of practical education and students’ agricultural entrepreneurial intentions.

H2b: Social status perception plays an intermediary role in influencing of practical education and students’ agricultural entrepreneurial intentions.

H2c: Professional value perception plays an intermediary role in influencing of practical education and students’ agricultural entrepreneurial intentions.

### 2.3. The influence of startup support policies on the students’ willingness to start agricultural businesses

Empowerment theory posits that by empowering individuals, their capacity to make proactive decisions and assume control over their actions is enhanced, ultimately resulting in behavioral modifications. Scholars have proposed that empowerment entails utilizing external strengths to assist individuals in gaining the power, resources, and skills required for implementing behaviors [35]. The final conscious generation of any individual behavior is intricately linked to the interplay of cognitive and situational factors [36]. In the context of young students’ agricultural entrepreneurship, in addition to their own family resources, entrepreneurial support policies should serve as an external empowering force that can help cultivate their perceptions of entrepreneurial efficacy and enable them to realize their potential. A wealth of research results have supported this view, indicating that students school support for entrepreneurship and motivation play a crucial role in enhancing students’ green entrepreneurial behavior [37]. Some research shows that the government’s policy mechanism to support entrepreneurship can positively promote successful entrepreneurship among returning students [38]. Based on the examination of 59 policy texts related to entrepreneurship for university students, some scholars have determined that these policies can empower and support students in their entrepreneurial endeavors, ultimately fostering sustainable development in university students entrepreneurship [28].

The following hypotheses are formed:

H3a: Entrepreneurship support policy positively moderates the effect of economic benefit perception on students’ willingness to start agricultural business.

H3b: Entrepreneurship support policy positively moderates the effect of social status perception on students’ willingness to start agricultural business.

H3c: Entrepreneurship support policy positively moderates the effect of professional value perception on students’ willingness to start agricultural business.

**Fig 1:**
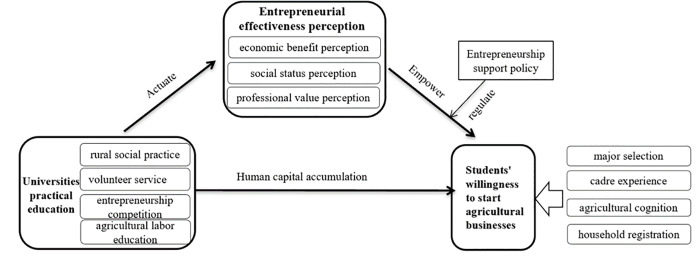
A theoretical model for analyzing students’ entrepreneurial intentions.

Accordingly, practical education can help enhance university students’ perceptions of the economic benefits, social status, and professional value of agricultural entrepreneurship. The three high levels of entrepreneurial effectiveness perception can further promote their willingness under the moderation of support policies. Therefore, this paper constructs a theoretical model to analyze students’ willingness to start agricultural businesses (Fig 1).

## 3. Materials and methods

### 3.1. Data sources

The data for this study came from the practical education survey conducted by the Sichuan Agricultural University from 20 January 2024–3 February 2024. This study passed the ethical review of the Youth Research Committee of Sichuan Agricultural University in January 2024. The documents showed that the research design was scientific, reasonable, fair and open, and no private data such as participants’ human health was involved, no harm was caused to participants, and there was no moral hazard. Participants were recruited based on the principle of voluntary and informed consent, and the rights and privacy of participants were protected. There is no conflict of interest or violation of ethical and legal prohibitions in the research content. Due to the vast territory of China, data in different regions may have heterogeneity. Therefore, to ensure the representativeness of the data samples, this study selected two agricultural universities respectively from the economically backward western and central regions and the economically developed eastern regions. To ensure that the samples are not subject to subjective selection, a stratified random sampling method is adopted. The sampling schools include: Sichuan Agricultural University and Northwest Agriculture and Forestry University in the western region, Henan Agricultural University and Huazhong Agricultural University in the central region, and Northeast Agricultural University and Nanjing Agricultural University in the eastern region. The investigation team distributed questionnaires through Internet communication tools and questionnaire platforms. On the premise of obtaining full informed consent, they encouraged students from the above-mentioned schools to fill out the questionnaires and participate in the survey. After statistical sorting, a total of 1,830 students participated in the survey, and 1,659 students completed the questionnaire filling, with a completion rate of 90.66%. After screening out invalid questionnaires, 1,612 valid questionnaires were finally obtained, with a questionnaire validity rate of 97.17%. The final survey sample had no response bias. The survey content includes details such as university practical education, students’ personal resources, perceived entrepreneurial efficiency, and their willingness to start agricultural enterprises.

### 3.2. Variable selection

The explanatory variable in this study is the willingness of students to start agricultural business, as determined by the questionnaire “Do you have the willingness to start agricultural business in the future? “ Characterization. The sample answers “yes” and “no” were assigned to 1 and 0, respectively. The statistical results show that 34.8% of the sample of universities have agricultural entrepreneurial intentions. This indicates that a portion of students in China’s agricultural universities have a career preference for agricultural entrepreneurship. Agriculture has gradually become an economic industry that absorbs youth employment.

The questionnaire identifies the explanatory variables of this study through four questions, such as ‘Do you take part in the university-organized social practice to the countryside?’ In a similar way, we found and evaluated the status of students’ participation in rural social practice, volunteer service, entrepreneurial competition, and agricultural labor education. Moreover, Refer to Thompson (2019) et al., and Luo Lei (2022) et al., multi-indicator accumulation to measure the overall degree [39,40], the cumulative total of the four sub-indicators was utilized to gauge the overall engagement of university students in practical education within higher education. The statistical data reveals that 75.9%, 84.7%, 77.4%, and 71.8% of the university students sampled engage in the four practical activities. On average, the sample participated in 3.099 practical education activities, indicating a high popularity of practical education in agricultural universities, with strong students acceptance.

In this study, the mediating variable being examined is the perception of entrepreneurial efficacy, which is further broken down into the perception of economic benefits, social status, and professional value within the context of agricultural entrepreneurship. The questionnaires used in the study included the following inquiries: “Do you believe that agricultural entrepreneurship offers significant economic benefits?”, “Do you believe that agricultural entrepreneurship is associated with high social status?”, and “Do you believe that agricultural entrepreneurship aligns with your personal interests and reflects your professional value?”. Responses to these questions were measured by assigning a value of 1 for “yes” answers and 0 for “no” answers from the sample population. The statistical findings reveal that 52.6% of the samples perceive economic benefits, while 24.6% have social status perceptions and 52.8% value the professional aspect of agricultural entrepreneurship. This suggests that Chinese students in agricultural universities predominantly identify with the professional value of agricultural entrepreneurship, with lower social status perceptions due to the geographical and labor-intensive challenges associated with agriculture.

The moderating variable under consideration is entrepreneurship support policies, with the questionnaire featuring the question “Are agricultural-related entrepreneurship support policies attractive to you?” In terms of characterization, the statistical analysis shows that the mean value of the sample support policies is 0.323, indicating that less than one-third of the students respondents find the current agricultural entrepreneurship support policies to be appealing. This shows the low level of support for agricultural entrepreneurship in China.

Referring to existing research [9,13,14], the controlled variables mainly include individual characteristics and family endowments. In this study, we control for sample political identity, major selection, students cadre experience, agricultural cognition, and 10 other variables as shown in Table 1.

**Table 1:** Meaning and assignment of variables.

Variable type	Variable		Assign a value	Averages	Standard deviation
Explained variable	Students’ willingness to start start agricultural businesses		Yes = 1; No = 0	0.348	0.476
Explanatory variable	Practical education	Rural social practice	Participation: Yes = 1; No = 0	0.759	0.428
		Volunteer service	Participation: Yes = 1; No = 0	0.847	0.360
		Entrepreneurship competition	Participation: Yes = 1; No = 0	0.774	0.418
		Agricultural labor education	Participation: Yes = 1; No = 0	0.718	0.450
		Total degree of practical education	Accumulating the four types of participating	3.099	1.188
Mediating variables	Entrepreneurial effectiveness perception	Economic benefit perception	Yes = 1; No = 0	0.526	0.499
		Social status perception	Yes = 1; No = 0	0.246	0.431
		Professional value perception	Yes = 1; No = 0	0.528	0.499
Adjustment variable	Entrepreneurial policy support		Entrepreneurship support policies are attractive: Yes = 1; No = 0	0.323	0.468
Control variable	Individual characteristics of respondents	Sex	Male = 0; Female = 1	0.616	0.487
		Political identity	Whether a member of the Communist Party of China: Yes = 1; No = 0	0.246	0.431
		Major selection	Whether to take the initiative to choose agriculture major: Yes = 1; No = 0	0.497	0.500
		Experience as a students cadre	Whether served as a students cadre: Yes = 1; No = 0	0.406	0.491
		Agricultural Cognition	Level of knowledge of the current situation in agricultural development: very low = 1; low = 2; average = 3; high = 4; very high = 5	3.236	0.856
		Agricultural correlation degree	Degree of connection between university education and agriculture: very low = 1; low = 2; average = 3; high = 4; very high = 5	4.102	0.968
	Family resource endowment	Household registration	Urban domicile = 0; rural domicile = 1	0.564	0.496
		Hometown countryside development	Level of rural development in home village: very low = 1; low = 2; average = 3; high = 4; very high = 5	3.320	0.775
		Family attitude	Parents’ attitude towards choice of agricultural employment: not supportive = 1; less supportive = 2; indifferent = 3; more supportive = 4; very supportive = 5	2.913	1.034
		Occupation of family members	The number of three-generation relatives working in agriculture	2.199	1.819

### 3.3. Research methods

To explore the impact of practical education on university students’ willingness to start agricultural businesses, we first construct the following regression model:


$$y_{i}=a_{i}+\beta_{\text{1}}x_{\text{1}}+\beta_{\text{2}}x_{\text{2}}+\beta_{\text{3}}x_{\text{3}}+\beta_{\text{4}}x_{\text{4}}+\varepsilon_{i}$$ (1)

In (1), a_i_ represents the intercept term, β represents the regression coefficient, y_i_ represents the agricultural entrepreneurial intention of the university students, ε_i_ represents the random variable, x1, x2, x3, x4 represent the practical education factors such as rural social practice, volunteer service, entrepreneurship competition and agricultural labor education, respectively. ε_i_ represents the random error term.

Since the dependent variable is a binary variable, it is advisable to employ a probit model for testing in order to mitigate any issues in model estimation [41]. Additionally, the empirical model is formulated as follows:


$$Prob(Y_{i}=\text{1}|x_{i})=Prob({\alpha_{\text{0}}education}_{i}+{\beta_{\text{0}}X}_{i}+\mu_{\text{0}})$$ (2)

(2) In the formula, Y_i_ is a binary discrete variable, Y_i_ = 1 indicates that university students have the intention of agricultural entrepreneurship, and vice versa, Y_i_ = 0; education_i_ indicates that the students received practical education in universities; X_i_ is a control variable; α_0_, β_0_ are the estimation coefficients; and μ_0_ indicates the random error term. In order to scientifically measure the above impact effects, this paper uses OLS and Probit models for regression analysis and validation respectively.

It is crucial to take into account the likelihood of endogeneity between university students’ participation in practical education and their desire to start agricultural businesses, as there is a common bi-directional causality that may lead to skewed results. Due to this reason, the study employed the instrumental variable method of correction,

Heckman (1978) constructed the IV-Probit model and adopted the two-stage regression method for the first time [42]. Firstly, the Oprobit model is used to regress the endogenous explanatory variables to exogenous explanatory variables, all control variables, and instrumental variables in order to obtain the fitted value $\hat{{education}_{i}}$ of ${education}_{i}$. The specific form is:


$$\hat{{education}_{i}}=\alpha +\delta T_{i}+{\gamma X_{i}}^{,}$$ (3)

In the above formula, ${X_{i}}^{,}$represents all exogenous explanatory and control variables in the common Oprobit regression model, including cooperative organizational support, individual characteristics, family endowment, and production environment. $\hat{{education}_{i}}$ is the fitted value of the instrumental variable $T_{i}$ and the explanatory variable ${X_{i}}^{,},$ and $\varepsilon_{i}$ is the residual.

In the second stage, Oprobit regression analysis is conducted on $Prob(Y_{i}=\text{1}|x_{i})$ for $\hat{{education}_{i}}$ and other exogenous explanatory and control variables ${X_{i}}^{,}$, and the unbiased estimation $\beta^{*}$ of β can be obtained. The two-stage model is shown as follows:


$$Prob(Y_{i}=\text{1}|x_{i})=Prob({\beta^{*}\hat{{education}_{i}}+{\eta X_{i}}^{,}+\varepsilon_{i}}_{\text{0}})$$ (4)

Choosing instrumental variables based on the endogenous explanatory variables rather than the disturbance term [32]. In this case, the instrumental variable used is whether the sample students receive actual economic support. The questionnaire item is “Have students actually received relevant economic subsidies in their education?” Assign 1 to the students’ answer “yes” and 0 to “No” respectively. Due to the strong resource dependence of practical education activities such as social practice, volunteer services, entrepreneurship competitions, and agricultural labor education in rural social practice, the practical support that students receive in terms of financial subsidies has a strong correlation with whether they ultimately receive practical education, but has no direct relationship with their entrepreneurial intentions. Meanwhile, in Table 2, we constructed two regression models to test respectively: The influence of instrumental variables on practical education and the influence of endogenous variables on entrepreneurial intention. The verification results of regression analysis show that instrumental variables have a strong correlation with explanatory variables, but no correlation with students’ intentions, supporting the above theoretical analysis. Meanwhile, this instrumental variable has passed the F-value test and is not a weak instrumental variable. Therefore, this variable is suitable to be used as an instrumental variable in this study.

**Table 2:** Instrumental variable testing.

Variable	Model (1) Probit Practical education	Model (2) Probit Willingness to start agricultural businesses
Actual economic support	0.252***(0.061)	0.009(0.254)
Sample size	1612	1612
F	17.31(0.000)	—
R^2^	0.011	—

To further validate that perceptions of entrepreneurial efficacy, such as economic benefit perception, social status perception, and professional value perception, serve as a mediating factor between practical education and university students’ readiness to start agricultural businesses, a model was created using the mediation effect test method [43]:


$$Y_{i}={\alpha_{\text{0}}education}_{i}+{\beta_{\text{0}}X}_{i}+\mu_{\text{0}}$$ (5)

$${cognition}_{i}=\alpha_{\text{2}}{education}_{i}+\beta_{\text{2}}X_{i}+\mu_{\text{2}\;\;\;\;\;\;\;}$$ (6)

$$Y_{i}=\alpha_{\text{3}}{education}_{i}+\beta_{\text{3}}{cogniti\text{on}}_{i}+X_{\text{0}}X_{i}+\mu_{\text{3}}\;$$ (7)

In equation (3), α_0_ represents the overall impact of practical education, such as rural social practice, volunteer service, entrepreneurship competition, and agricultural labor education, and other practical education, on university students’ willingness to start agricultural businesses. In equation (4), α_2_ signifies the influence of practical education on the mediator variable, and cognition_i_ refers to the entrepreneurial effectiveness perception of sample i. In equation (5), α_3_ and β_3_ indicate the direct impact of practical education and entrepreneurial effectiveness perception on the entrepreneurial willingness of sample i.By substituting Eq. (4) into Eq. (5), we can derive the mediating effect α_2_β_3_. This mediating effect signifies the indirect influence of practical education on entrepreneurial willingness through entrepreneurial effectiveness perception within the sample. The mediating effect can be expressed as a percentage of α_2_β_3_/α_0_.

## 4. Results and discussion

### 4.1. Main effects analysis

Before the empirical analysis, we first conducted a reliability and validity analysis of the data. Using SPSS26.0 software, we verified that the alpha of the survey data (n = 1612) was 0.725 and the KMO value was 0.734. Both values were greater than 0.7, indicating that they have good reliability and validity. Applicable to empirical analysis. Meanwhile, collinearity diagnosis was conducted using STATA 14.0, and the VIF indicators of all indicators were greater than 1, indicating that there was no multicollinearity issue in all data.

As shown in Table 3, model (1)(2) uses OLS and Probit models, respectively, to examine the impact of university practical education, such as rural social practice, volunteer service, entrepreneurship competition, agricultural labor education, on the sample university students’ willingness to start agricultural businesses, controlling for individual major selection, experience as a student cadre and agricultural cognition, among other confounding factors. The results demonstrate that all four types of practical education have a significant, positive influence on students’ inclination towards agricultural entrepreneurship. These effects are statistically significant at the 5%−10% level, with the entrepreneurship competition having the highest and most significant coefficients. This indicates that university students who have participated in entrepreneurship competitions in universities are 22.0% more likely to be willing to start agricultural businesses than other groups of students. Models (3)(4), using OLS and Probit models respectively, found that the total amount of practical education was positively significant at the 1% level on agricultural entrepreneurial intentions of the sampled students. The results indicate that the richer the practical education in universities, the more likely students are to develop agricultural entrepreneurial intentions.

**Table 3:** Effect of practical education on students’ willingness to start agricultural holding.

Variable	Model (1) OLS	Model(2) Probit	Model (3) OLS	Model (4) Probit	Model (5) IV-Probit	Model(6) Marginal effect
Rural social practice	0.052*(0.031)	0.155*(0.090)	—	—	—	—
Volunteer service	0.050(0.038)	0.184*(0.111)	—	—	—	—
Entrepreneurship competition	0.071** (0.032)	0.220** (0.093)	—	—	—	—
Agricultural labor education	0.052* (0.030)	0.144* (0.086)	—	—	—	—
Total degree of practical education	—	—	0.056*** (0.011)	0.174*** (0.034)	0.318*** (0.081)	0.110*** (0.026)
Sex	−0.006 (0.024)	−0.019 (0.069)	−0.006 (0.024)	−0.017 (0.069)	−0.032 (0.069)	−0.011 (0.024)
Political identity	−0.038 (0.027)	−0.102 (0.078)	−0.038 (0.027)	−0.101 (0.078)	−0.083 (0.078)	−0.029 (0.027)
Major selection	0.051** (0.024)	0.146** (0.066)	0.051** (0.024)	0.146** (0.066)	0.118* (0.068)	0.041* (0.023)
Experience as a students cadre	0.055** (0.024)	0.156** (0.068)	0.054** (0.024)	0.155** (0.068)	0.164** (0.068)	0.057** (0.023)
Agricultural Cognition	0.029* (0.015)	0.095** (0.044)	0.029* (0.015)	0.095** (0.044)	0.076* (0.045)	0.026* (0.016)
Agricultural correlation degree	−0.015 (0.013)	−0.032 (0.039)	−0.016 (0.013)	−0.032 (0.039)	−0.068 (0.043)	−0.023 (0.015)
Household registration	0.057** (0.026)	0.167** (0.072)	0.057** (0.026)	0.167** (0.072)	0.136* (0.074)	0.047* (0.026)
Hometown countryside development	0.016 (0.016)	0.041 (0.045)	0.016 (0.016)	0.041 (0.045)	0.042 (0.045)	0.014 (0.015)
Family attitude	0.012 (0.013)	0.037 (0.036)	0.012 (0.013)	0.036 (0.036)	−0.023 (0.047)	−0.008 (0.016)
Occupation of family members	0.017*** (0.007)	0.049*** (0.019)	0.017*** (0.007)	0.049*** (0.019)	0.052*** (0.019)	0.018*** (0.007)
Sample size	1612	1612	1612	1612	1612	1612
Adjustment of R2/pseudo R^2^/lnsig	0.048	0.047	0.050	0.047	−0.064*** (0.000)	—
LRchi^2^/F	6.79	97.67 (0.000)	8.63	97.31 (0.000)	86.89 (0.000)	—
Log likelihood	—	−992.856	—	−993.033	−3174.621	—

Note: *** and ** denote 1% and 5% significance levels respectively; figures in brackets are robust standard errors of the coefficients; data in the table are rounded. The following is the same.

Next, (5) presents the estimation results using the IV-Probit model, with a significant lnsig value at the 1% level. Furthermore, through likelihood ratio testing, the two-stage estimation is significant, indicating that the estimation results are more accurate when using the IV-Probit model. Controlling for the issue of bidirectional endogeneity, the coefficient of practical education on agricultural entrepreneurship intention among university students is 0.318, with significance level consistent with the estimation results of model (4). This further confirms the positive impact of practical education on university students’ intention for agricultural entrepreneurship. Model (6) confirms Hypothesis 1 by showing that the likelihood of a university students being willing to start agricultural entrepreneurship increases by 11.0% for every additional practical education they receive, even after accounting for endogeneity concerns.

In terms of other control variables, the major selection, students cadres experience, agricultural awareness, household registration, and family members’ occupations all have an impact on the agricultural entrepreneurial willingness of the sampled university students. Specifically, the positive impact of students’ choice of major is significant at the 10% level, with students actively choosing agriculture as a major, proving that they are more interested in agriculture itself and more likely to follow their initial ideals into the agricultural business after they graduate. Youth with experience as students cadres are 5.7% more likely to be willing to start agricultural businesses than other students groups. Students cadre groups tend to have stronger creative skills and all-round qualities, are more likely to participate in activities such as social practice, voluntary service and entrepreneurship competitions, and are more likely to start a business in their own field of study after graduation. Agricultural awareness influences their entrepreneurial intention at a 10% level positively, possibly because awareness is the driving force behind behavioral willingness. The higher the level of understanding of agricultural development among students, the more likely they are to recognize the valuable window of opportunity for agricultural entrepreneurship under the current background of rural revitalization, and thus convert it into a willingness to start agricultural businesses. Rural registered university students are 4.7% more likely to be willing to start agricultural businesses compared to urban populations. Individuals who have previously lived in rural areas are more familiar with agriculture and are more likely to possess a sense of returning to their roots, unlike urban students who have had no long-term exposure to agriculture and rural areas during their upbringing. Alternatively, students from rural backgrounds may find themselves at a social and resource disadvantage, leading them to opt for the less competitive agricultural sector in the face of urban job competition. This scenario could be seen as a reflection of the current employment landscape. The influence of family members’ occupations on students’ willingness to start agricultural businesses is significantly positive at the 1% level. The more relatives in three generations of a young person’s family work in the agricultural industry, the more developed their agricultural business network becomes, increasing the likelihood of them being empowered to generate entrepreneurial aspirations due to their exceptional resource advantage.

### 4.2. The mediating effect of economic benefit perception and the moderating role of entrepreneurial support policies

Theoretical analyses indicate that individuals’ views on the economic advantages of agricultural entrepreneurship could partially mediate the impact of practical education in higher education on their entrepreneurial intentions. To empirically test this hypothesis and understand the underlying transmission mechanisms, stepwise regression analyses were conducted. As shown in Table 4, Model (1) uses probit estimation to examine the impact of practical education on students’ perception of economic benefits in agriculture entrepreneurship. The results indicate that practical education has a positive effect on the economic benefit perception of university students in agriculture entrepreneurship at the 1% significance level. The model (2) mainly focuses on the latter part of the mediating effect, that is, the impact of economic benefit perception on university students’ willingness to start agricultural businesses. The results show that this coefficient is 0.464, also a significant positive impact at the 1% level, indicating that the greater economic benefits university students perceive from agricultural entrepreneurship, the more likely they are to be willing to start a business due to potential benefits. Model (3) simultaneously introduces both independent and mediating variables. The results indicate that the estimated coefficients of practical education received by university students and their economic benefit perception are positive, and their impacts on the dependent variable are both significant at the 1% level. The estimates using the IV-Probit model are shown in column (4), with a ln(sig) value of −0.067, which is significant at the 1% level. The two-stage estimation is also significant, controlling for the endogeneity issues between the independent and dependent variables. The results remain consistent with the previous findings, showing that practical education in universities and economic benefit perception for students both have a positive impact on their willingness to start agricultural businesses. Compared to the IV-Probit estimate coefficient of 0.318 for practical education without introducing the perception of economic benefits, the impact coefficient of 0.297 on the sample agricultural entrepreneurial intention is lower, indicating that university students’ perception of the economic benefits of agricultural entrepreneurship plays a partial mediating role.

**Table 4:** Mediating effects of economic benefit perception and testing the moderating role of support policies.

Variable	Model (1) Probit Economic benefit perception	Model (2) Probit Willingness to start agricultural businesses	Model (3) Probit Willingness to start agricultural businesses	Model (4) IV-Probit Willingness to start agricultural businesses	Model (5) Probit Willingness to start agricultural businesses
Practical education	0.104*** (0.031)	—	0.162*** (0.034)	0.297*** (0.083)	—
Economic benefit perception	—	0.464*** (0.068)	0.444*** (0.068)	0.417*** (0.070)	0.346*** (0.084)
Entrepreneurship support policy	—	—	—	—	0.207* (0.108)
Economic benefit perception* Support Policies	—	—	—	—	0.312** (0.142)
Control variable	Control	Control	Control	Control	Control
Sample size	1612	1612	1612	1612	1612
LRchi^2^/Wald chi^2^	91.55 (0.000)	117.37 (0.000)	140.58 (0.000)	129.06 (0.000)	152.57 (0.000)
Pseudo R^2^/lnsig	0.041	0.056	0.068	−0.067*** (0.018)	0.073
Loglikelihood	−12069.391	−983.002	−971.399	−3149.938	−965.402

Synthesising the results of the calculations in models (1)-(4), it is easy to see that individual perceptions of economic benefits play a partially mediating role in the process of practical education influencing students’ willingness to start agricultural businesses. Specifically, with the enrichment of practical education in universities, students’ personal perception and practical practice in activities such as social practice in the rural social practice, volunteer service, entrepreneurship competition, and agricultural labor education, and the level of perception of the economic benefits of agricultural entrepreneurship increase, leading them to develop the intention of agricultural entrepreneurship, and hypothesis H2a is confirmed.

To test the hypothesis that entrepreneurial support policies play a moderating role in the second half of the mediating effect, model (5) simultaneously introduces economic benefit perception, agricultural entrepreneurial support policies, and their interaction terms. Using Probit for empirical calculations, the results show that economic benefit perception and support policies have a significant positive impact on university students’ entrepreneurial intentions at the 1% and 10% levels, respectively. The interaction term is also positively significant at the 5% level, indicating that agricultural entrepreneurial support policies play a positive moderating role in the process of economic benefit perception affecting the entrepreneurial intentions of the sample. Hypothesis H3a is confirmed. When university students have a high perception of the economic benefits of agricultural entrepreneurship, and when government departments have stronger support policies for youth agricultural entrepreneurship, young university students, as rational economic individuals, are more likely to have the psychological foundation and entrepreneurial confidence supported by agricultural and rural policies. This, in turn, can stimulate their spirit of hard work and entrepreneurial awareness. Therefore, entrepreneurial support policies can positively adjust the impact of economic benefit perception on students’ willingness to start agricultural businesses.

### 4.3. The mediating effect of social status perception and the moderating role of entrepreneurial support policies

To assess the hypothesis that the social status perception of students in relation to agricultural entrepreneurship partially mediates the effect of practical education on their entrepreneurial intentions, probit and IV-probit models were utilized. As shown in Table 5, the computational results of models (1)-(3) indicate that the effect of practical education on the sample’s perception of social status in agricultural entrepreneurship is significantly positive. Under the catalysis of campus practical education, if students believe that engaging in agricultural entrepreneurship can help them identify their self-worth in social activities and consider their entrepreneurial activities meaningful and valuable, they are more likely to have the actual intention to start a business. That is, their perception of social status plays a partial mediating role in the influence of practical education on promoting their agricultural entrepreneurship. Specifically, as universities enhance the richness of practical education, the perception of self-social status improvement brought by agricultural entrepreneurship activities changes accordingly among education recipients. university students are more likely to develop self-value recognition and social achievement recognition towards agricultural entrepreneurship, thus becoming willing to cultivate agricultural entrepreneurship intentions. After addressing endogeneity concerns, the results from the IV-Probit model in column (4) reaffirm the positive influence of practical education in higher education institutions and economic benefit perception on agricultural entrepreneurial intentions. Additionally, it confirms the partial mediating role of social status perception, providing further evidence for hypothesis H2b.

**Table 5:** Moderating effects of social status perception and the moderating role of support policy test.

Variable	Model (1) Probit social status perception	Model (2) Probit Willingness to start agricultural businesses	Model (3) Probit Willingness to start agricultural businesses	Model (4) IV-Probit Willingness to start agricultural businesses	Model (5) Probit Willingness to start agricultural businesses
Practical education	0.132*** (0.036)	—	0.153*** (0.034)	0.265*** (0.084)	—
Social status perception	—	0.703*** (0.077)	0.680*** (0.077)	0.654*** (0.080)	0.558*** (0.098)
Entrepreneurship support policy	—	—	—	—	0.265*** (0.084)
Social status perception* Support policies	—	—	—	—	0.301* (0.158)
Control variable	Control	Control	Control	Control	Control
Sample size	1612	1612	1612	1612	1612
LRchi^2^/Wald chi^2^	108.65 (0.000)	154.51 (0.000)	175.40 (0.000)	161.57 (0.000)	182.56 (0.000)
Pseudo R^2^/lnsig	0.060	0.074	0.084	−0.066*** (0.018)	0.088
Loglikelihood	−844.388	−964.433	−953.991	−3134.376	−950.410

Model (5) introduces both social status perception and agricultural entrepreneurship support policies, as well as their interaction terms. The results show that both social status perceptions and support policies positively affect university students’ agricultural entrepreneurial intentions at the 1% significant level, and the interaction term is positively significant at the 10% level, indicating that agricultural entrepreneurial support policies positively moderated the effect of social status perceptions on their entrepreneurial intentions. This can be explained by the fact that when students have a strong desire for social status in the field of agricultural entrepreneurship, the more supportive policies there are for youth agricultural entrepreneurship, the more likely it is that they will feel that social status in this field is attainable. This, in turn, can positively influence their motivation to pursue entrepreneurship and lead to the belief that support policies can help them succeed in high-quality agricultural entrepreneurship and attain higher social status after graduation. This hypothesis (H3b) was confirmed.

### 4.4. The mediating effect of professional value perception and the moderating role of entrepreneurship policies

As with previous outcomes of this work, as indicated in Table 6, models (1)-(4) validated the beneficial impact of practical education on students’ professional value perception, the favorable effect of an individual’s professional value perception in agricultural entrepreneurship on their entrepreneurial intention, and the mediating role played by students’ professional value perception in this process. As practical education in universities continues to expand, the perception of the professional value of agricultural entrepreneurship among individuals is also increasing. University students are becoming more open to the idea that agribusiness entrepreneurship allows them to apply their professional knowledge and pursue their interests. This increased acceptance may lead to a higher likelihood of students being willing to start a business in the agricultural sector, thus confirming hypothesis H2c. In model (5), both professional value perception, support policies and their interaction terms are introduced. The study found that professional value perception and support policies had a positive and significant impact on university students’ intentions to start agricultural businesses. However, the interaction term was not significant, indicating that hypothesis H3c was not supported. This could be because professional value perception is influenced more by individual interests and subjective judgments, leading students to make choices based on their own motivations. Additionally, entrepreneurship support policies that focus on material incentives may struggle to change the career orientation of young students who prioritize their individual interests.

**Table 6:** Mediating effects of professional value perception and the moderating role of support policy test.

Variable	Model (1) Probit Professional value perception	Model (2) Probit Willingness to start agricultural businesses	Model (3) Probit Willingness to start agricultural businesses	Model (4) IV-Probit Willingness to start agricultural businesses	Model (5) Probit Willingness to start agricultural businesses
Practical education	0.112*** (0.032)	—	0.153*** (0.034)	0.280*** (0.084)	—
Professional value perception	—	0.625*** (0.069)	0.602*** (0.069)	0.574*** (0.072)	0.584*** (0.086)
Entrepreneurship support policy	—	—	—	—	0.336*** (0.108)
Professional value perception* Support policy	—	—	—	—	0.188 (0.143)
Control variable	Control	Control	Control	Control	Control
Sample size	1612	1612	1612	1612	1612
LRchi^2^/Wald chi^2^	123.45 (0.000)	153.58 (0.000)	174.32 (0.000)	161.84 (0.000)	193.87 (0.000)
Pseudo R^2^/lnsig	0.055	0.074	0.084	−0.067*** (0.018)	0.093
Loglikelihood	−1053.115	−964.900	−954.531	−3133.161	−944.755

### 4.5. Robustness testing

On the one hand, the main effects model was tested for model robustness. Replacing the main effects model with a Logit model, the impact of practical education in universities on university students’ willingness to start agricultural businesses remains significant at the 1% level, indicating good model robustness.

On the other hand, the mediated effects model was tested for model robustness. Replacement of the mediation effect test was tested using the Sobel and Bootstrap methods [44]. As indicated in Table 7, the results indicate that students’ perceptions of the economic benefits, social status, and professional value of agricultural entrepreneurship all showed significant mediating effects at the 1% level. This means that these three types of entrepreneurial effectiveness perception partially mediate the main effects, with mediation effect values of 0.006, 0.008, and 0.009, accounting for 10.7%, 14.3%, and 16.1% respectively. This confirms the robustness of the three mediating mechanisms and reveals that the perception of professional value plays the largest mediating role in influencing university students’ willingness to start agricultural businesses through practical education in universities.

**Table 7:** Bootstrap test for mediation effects.

	Total effect	Intermediary effect	Proportion	Standard deviation	Lower limit LLCI	Upper limit ULCI
Economic benefit perception	0.056	0.006	0.107	0.002	0.002	0.010
Social status perception	0.056	0.008	0.143	0.003	0.003	0.013
Professional value perception	0.056	0.009	0.161	0.003	0.003	0.014

### 4.6. Discussion

The empirical results of this study show that practical education in agricultural universities can effectively promote students’ employment in the agricultural sector [45,46], establish agricultural enterprises [47,48], and play an important role in shaping individual agricultural entrepreneurship and employment intentions [26]. In the research on individual behavior, we also used rational economic agents as the core basis for behavioral decisions [49]. Through the verification of survey data, we also found that personal characteristic variables such as students’ professional choices, agricultural cognition, and household registration also have an impact on individual behavior, which is consistent with the existing research conclusions [12,13]. This study also reveals that agricultural universities, as the main hub of talent training for modern agricultural development, play a vital role in promoting social development, rural prosperity and farmers’ economic well-being [5,50]. University practical education needs to be offered in multiple dimensions [29], which can play an important role in shaping students’ values, cultivating innovation and entrepreneurship, and promoting the consciousness of serving the society [6–8], and help them cultivate the perception of agricultural entrepreneurial value.

However, unlike previous studies, the dimensions for measuring practical education in universities in existing research are very limited. Some scholars only conduct research from the perspective of spiritual and policy education [51], while others only study from the perspective of entrepreneurship education [31]. This study has established a very sound measurement system, innovatively measuring university practical education from four dimensions: knowledge explanation, entrepreneurship competition, labor education and practical activities, which is more systematic and comprehensive. In addition, we found the influence mechanism of practical education promoting students to establish agricultural enterprises, which is different from entrepreneurship [47] and agricultural attitude [52] as mediating variables proposed in previous studies. Some studies suggest that the mediating variables in this influence are entrepreneurial ability [53] and entrepreneurial spirit [54], which is not comprehensive enough. Based on the research of Wu et al (2022) [31] and Saoula et al (2023) [32], we propose that entrepreneurial efficacy is a mediating variable of this influence. However, our measurement of perception is more systematic than their, and we measure and explore it from the perspectives of economic value, social value and professional value. It provides new research ideas for future agricultural entrepreneurship research and the enrichment system of research indicators. This study also innovatively found that entrepreneurship support policies play a role in regulating the connection among individual economic benefits, perception of social status and willingness. However, it cannot regulate the impact of professional value perception on the willingness to start an agricultural business. This first discovery may be due to the fact that professional value perception is closely related to the characteristics of the agricultural industry. In the analysis of individual behavior, the influence of learning scenarios is subtle and cannot be ignored [55]. The group of students has a special self-awareness ability [56]. Students’ perception of the value of their major stems from long-term learning accumulation and the formation of understanding. This is a long-term and individual autonomous system that is not easily disturbed or influenced by the outside world. Therefore, even if strong entrepreneurial support policies are provided to individuals, it cannot change students’ views on the value of their own majors.

The long-term impact of practical education on the success of agricultural enterprises is that it can promote the initial possibility of enterprise establishment. The implementation of university practical education enables students to accumulate confidence and multiple expectations for the establishment of agricultural enterprises in the process of classroom learning, entrepreneurial competition, agricultural labor and other practices, and encourages them to dare to take the first step of establishing agricultural enterprises. This is the most basic and necessary condition for business growth. Therefore, through the development of university practice education, it can promote the sustainable development of talents in agricultural enterprises.

## 5. Conclusions and recommendations

The impact and mechanism of practical education in universities, students’ entrepreneurial effectiveness perception, and agricultural entrepreneurial intentions were systematically explained and empirically tested in this study. It was discovered that the implementation of rural social practice, volunteer service, entrepreneurship competition, and agricultural labor education by universities can significantly boost university students’ interest in starting their agricultural businesses. Additionally, for every additional type of practical education students receive, they are 11.0% more likely to want to start business in agriculture. Practical education will affect individual entrepreneurial effectiveness perception, including economic benefit perception, social status perception and professional value perception. Among these, the mediating effect of professional value perception is the strongest, with a ratio of 16.1%. Entrepreneurship support policy play a role in moderating the connection between individuals’ economic benefit, social status perceptions and their intentions. The intentions of students to start agricultural businesses are significantly correlated with their control variables, including major selection, students cadre experience, agricultural cognition, household registration, and family members’ occupation. Therefore, it is suggested that there should be a focus on enhancing practical education in agricultural universities, fostering students’ confidence in agricultural entrepreneurship, and optimizing policy support for university students’ entrepreneurial endeavors.

Accordingly, the following policy recommendations are made:

Firstly, agricultural universities should strengthen practical education, promote activities such as rural social practice, volunteer service, entrepreneurship competition, and agricultural labor education to penetrate deeper and deeper into the hearts of young students, highlight the strategic position of the rural areas, and organically combine the elements of agriculture and rural areas with practical education, so as to encourage the young generation to closely cultivate their feelings for the rural areas, and to enhance their sense of identity and honour in the agricultural industry.

Secondly, students should be encouraged to enhance their perception of entrepreneurial effectiveness. By regularly holding mobile lectures and classroom teaching on agricultural entrepreneurship, we help them understand the economic benefits, social impacts and professional values of agricultural entrepreneurship, especially focusing on guiding them to recognize the professional value of agricultural entrepreneurship, maximizing the intermediary effect to guide their entrepreneurship, and inspiring them to pursue entrepreneurial opportunities with enthusiasm.

Thirdly, it is important to encourage and guide graduates from agricultural colleges to start businesses in the agricultural sector through policy support. Initiatives such as compensating students tuition fees, national students loan repayments, agricultural entrepreneurship rewards, project subsidies, providing entrepreneurship practice bases and internships, can empower them to achieve successful entrepreneurial outcomes and support the implementation of their business activities.

Fourthly, practical education in university should be tailored to the needs of students. By paying attention to the various students groups with different backgrounds in major selection, students cadreship experience, agricultural knowledge, place of origin, and family members’ professions, we may be able to tailor our practical education strategies to better meet the unique needs of each group. Our focus is on guiding and nurturing students who have a strong interest in agriculture, possess high levels of overall competence and skills, have a comprehensive understanding of sustainable development, and hail from rural areas. To support these students, increasing funding for entrepreneurship, providing more opportunities for rural internships and training, and establishing mentorship programs to help them succeed in their agricultural business ventures.

## Supporting information

S1 File Data. (XLSX)
