# Expression of Concern: Promoting agricultural entrepreneurship through practical education in Chinese universities

**DOI:** 10.1371/journal.pone.0360711

**Published:** 2026-10-15

**Authors:** The PLOS One Editors

After this article [1] was published, the following concerns were noted:

- The articles cited as References 21, 30, 35, 36, and 39 do not appear to support the corresponding cited statements.
- The articles cited as References 29, 54, and 56 were introduced without editorial approval, and appear to be irrelevant to the article and corresponding cited statements.

In light of the cumulative issues, the *PLOS One* Editors issue this Expression of Concern. Readers are advised to interpret the article [1] with caution.
